# Functional Brain Asymmetry and Menopausal Treatments: Is There a Link?

**DOI:** 10.3390/medicina58050616

**Published:** 2022-04-28

**Authors:** Giuseppe Alessandro Digesu, Gaetano Riemma, Marco Torella, Marco La Verde, Antonio Schiattarella, Gaetano Maria Munno, Diego Domenico Fasulo, Angela Celardo, Primo Vagnetti, Salvatore Annona, Maria Teresa Schettino, Maurizio Guida, Pasquale De Franciscis

**Affiliations:** 1 St Mary’s Hospital, Imperial College Healthcare Trust, London OX3-01865, UK; 2 Obstetrics and Gynecology Unit, Department of Woman, Child and General and Specialized Surgery, University of Campania “Luigi Vanvitelli”, 80138 Naples, Italy; gaetano.riemma@unicampania.it (G.R.); marcotorella@iol.it (M.T.); marco.laverde88@gmail.com (M.L.V.); aschiattarella@gmail.com (A.S.); gmm9401@gmail.com (G.M.M.); diegodomenico1993@gmail.com (D.D.F.); angelacelardo@gmail.com (A.C.); primo.vagnetti@unicampania.it (P.V.); studio@annona.it (S.A.); mariateresa.sche@gmail.com (M.T.S.); pasquale.defranciscis@unicampania.it (P.D.F.); 3 School of Medicine, Department of Neuroscience, Reproductive Sciences and Dentistry, University of Naples Federico II, 80131 Naples, Italy; maurizio.guida@unina.it

**Keywords:** functional cerebral asymmetry, menopause, HRT, MHT, soy isoflavones, phytoestrogens

## Abstract

*Background and Objectives*: The human brain presents a functional asymmetry for every cognitive function, and it is possible that sexual hormones could have an impact on it. Visual–spatial attention, one of the most lateralized functions and one that is mainly dependent on the right hemisphere, represents a sentinel for functional cerebral asymmetry (FCA). The aim of this study was to evaluate whether menopausal hormone therapy (MHT) or phytoestrogens could modulate FCA in postmenopausal women. *Materials and Methods*: We enrolled postmenopausal women who were taking MHT or soy isoflavones or receiving no therapy and asked them to perform the line bisection test at study enrollment and after 18 and 36 months. *Results*: Ninety women completed the follow-up. At zero time, women who had not been subjected to therapy showed a leftward deviation (F = −3.0), whereas, after 36 months, the test results showed a rightward deviation (F = 4.5; *p* < 0.01). Women taking MHT showed a leftward deviation at the start (F = −3.0) and a persistent leftward deviation after 36 months (F = −4.0; *p* = 0.08). Conversely, women taking soy isoflavones started with a leftward deviation (F = −3.0) that became rightward (F = 3.0), with a significant difference shown after 36 months (*p* < 0.01). *Conclusions*: Our data suggest that hormonal modulation improves the interplay between the two hemispheres and reduces FCA. We propose, therefore, that the functions of the right hemisphere are mainly affected by aging and that this could be one of the reasons why the right hemisphere is more susceptible to the effects of MHT.

## 1. Introduction

In the human brain, there is a functional asymmetry for every cognitive ability that relates to the dominance of one or the other hemisphere [1,2,3]. This condition is defined as right or left brain dominance and can differ between women and men. Cerebral lateralization represents a crucial aspect of human brain organization: The right hemisphere functions include spatial cognition, language, and face recognition, and the left hemisphere specializes in linguistic processes, sequential processes, the logical concatenation of thought, the management of the cause–effect relationship, and the analytical perception of reality [4,5].

Evidence shows that sexual hormones can permanently influence brain functions and human behavior, including functional cerebral asymmetry (FCA). Progesterone seems to influence the glutamatergic synaptic transmission through decreased activity, but it also reduces cortico–cortical transmission, decouples the hemispheres, and reduces the extent of functional asymmetries [6,7]. On the other hand, the role of estradiol regarding the impact on left–right differences has not yet been clearly understood [1,8]. Menopause represents an ideal model for expanding knowledge on the effects of hormone modulations on FCA because of the physiological absence of estrogens and progesterone. Sexual hormones act through two mechanisms: reducing the activation of the right or left motor system, thus reducing the disparity between the two hemispheres, or modulating the interactions between the dominant right hemisphere and the motor areas of the left hemisphere [6,9,10].

Menopause hormonal therapy (MHT) represents the gold standard for the treatment of menopausal disorders. Several studies have revealed the presence of estrogen receptors in multiple areas of the brain (e.g., striated body, limbic system, cortex) involved in cognitive processes, such as language, memory, and spatial skills [6,9]. Recent data acquired throughout imaging techniques suggest that MHT modulates the functional organization of the cortex during the execution of cognitive activities and seems to protect against cognitive decline and the development of Alzheimer’s disease [11]. 

Even if MHT is a first-line therapy, some women have multiple contraindications, experience side effects, are noncompliant with the treatment, or simply refuse to start MHT, asking for a “natural” approach [12,13,14,15,16]. 

Nutraceuticals have been introduced as alternative compounds for the management of menopausal discomforts. As dietary supplements, they are administered to peri- and postmenopausal patients [7,8] since they can improve vasomotor symptoms, as supported by growing evidence; however, definitive data regarding their safety are lacking.

Soy isoflavones are among the most common nutraceutical compounds for menopausal complaints as they act as weak estrogen receptor (ER) agonists [12,13]. They show about seven-fold lower affinity for ERs than that of estradiol, but hypoestrogenism is sufficient to cause biological activity called paraestrogenic effects [17]. However, there are currently no data on their effects on brain function. 

One of the tests used to evaluate the disturbances of the visuospatial attention is the line bisection test, known as Bisiach’s test, which consists of analyzing the bisection point of a series of straight lines [18]. The direction of the midpoint is a function of the hand being used in that the left hand corresponds to the right space and vice versa [4]. However, the left hemisphere controls only cognitive functions in the contralateral right hemispace, whereas the right hemisphere manages both sides of space with the dominance of the contralateral side [3,4]. This suggests that hemispheric asymmetry controls the hand effect such that the hemispheres overlap and the superiority of the right hemisphere is present in visual–spatial attention for both sides [1,3,19]. Healthy fertile women present less asymmetry between the two hemispheres when steroid hormone levels are higher, such as during the luteal phase, and their hand use effect is reduced [1,10]. In healthy women, the midpoint falls mostly in the left half, regardless of the hand used, probably due to a more efficient inter-hemispheric connection than in men, in whom the posterior portion of the corpus callosum is assumed to be less expressed [20,21]. To date, the definite role of estrogens and progestins in the modulation of interhemispheric connections is still unclear. 

The objective of our study is to evaluate whether MHT and soy isoflavones modulate FCA in postmenopausal women. 

## 2. Materials and Methods

This was a prospective observational study performed at the Unit of Obstetrics and Gynecology, AOU Luigi Vanvitelli at the University of Campania Luigi Vanvitelli in Naples, Italy. The study was performed in accordance with the principles of the Helsinki Declaration. As the study was classified as a hospital audit of current clinical practice, the need for ethical approval was waived by the institutional review board (IRB) of the university hospital. All participants signed a written informed consent form before enrollment. The anonymity and privacy of all participants were maintained in every part of the study.

We included consecutive postmenopausal women who attended our outpatient menopause clinic. The patients were divided into three groups according to the treatment prescribed by their personal gynecologist or by the gynecologist of the center: no treatment (group A), continuous combined MHT (group B), or soy isoflavones (group C). 

The continuous combined MHT contained 1 mg of estradiol hemihydrate and 2 mg of drospirenone; soy isoflavones contained 30 mg of genistein and 30 mg of daidzin obtained from purified and titrated extracts of soybeans (*Glycine max* L.) that were not genetically modified (GMO-free); the extracts were purified from inert components to obtain a concentration of isofavones of 40%.

We excluded from the analysis women determined to be left-handed by the Edinburgh Handedness Inventory [22]; women with abnormal or incorrect visual acuity; women with neurological or psychiatric disorders who had used drugs that could affect the central nervous system during the six months prior to the study; and women who were obese (BMI > 30 kg/m^2^). We excluded from the analysis women undergoing therapy that had started more than 30 days before; women who were following a soy-enriched diet; women who had received estrogen therapy in the last six months; smokers; women with anamnestic or ongoing neoplastic disease, arterial hypertension, anemia, or endocrine pathology (diabetes, thyroid diseases, cortico-adrenal changes); and women who were receiving sequential MHT. 

The line bisection test, also known as Bisiach’s test, was administered at study enrollment and after 18 and 36 months. The test was performed at a table in a quiet private room, with the experimenter sitting directly opposite the patient. The test consisted of three black horizontal lines of one millimeter thickness, ranging in length from 100 mm to 200 mm, drawn on a white sheet of paper. The patients were asked to divide each line into two parts of equal length by marking the subjective midpoint of each line with a fine pencil, using first the dominant hand (right) and then the other. The other lines were covered so as not to influence the next choice. There were no time restrictions. The deviation left or right from the effective half for each line was measured with an accuracy of 0.5 mm. 

The study followed the STROBE (strengthening the reporting of the observational studies in epidemiology) guidelines, available through the EQUATOR (Enhancing the QUAlity and Transparency Of health Research) network (http://www.equatornetwork.org/, accessed on 7 March 2022) [23].

Data were reported as means ± standard deviation (SD) for continuous variables or as numbers (percentages) for dichotomous variables. Comparisons between groups were assessed with the Pearson chi-square test and Fisher’s exact test for categorical variables and Student’s *t*-test or analysis of variance (ANOVA) for continuous variables. Multiple logistic regression was performed to identify covariates that were associated with the changes in the primary outcome of interest. A *p*-value < 0.05 was considered statistically significant. Statistical analysis was performed using SPSS for Windows (version 15.0, SPSS, Chicago, IL, USA). 

## 3. Results

**Figure 1:**
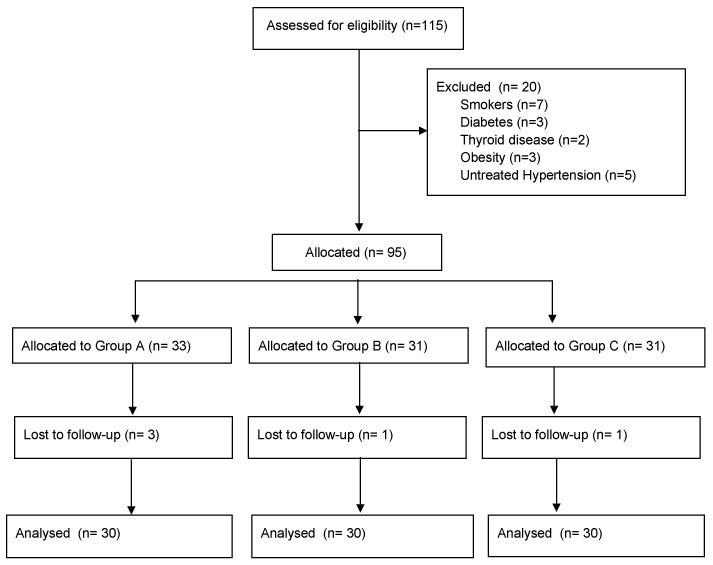
STROBE flow-chart of patients included in the analysis.

A total of 115 women were considered eligible for the study, and, after the application of the exclusion criteria, 20 patients were excluded (Figure 1). Five patients were lost during follow-up. Thus, ninety women (30 for each group) completed the follow-up and were eligible for data analysis.

The mean age of the participants was 53.5 ± 2.4 years. All women were Caucasian. The groups showed no difference in age, level of education, or years after menopause. The main characteristics of the study population are shown in Table 1.

**Figure 2:**
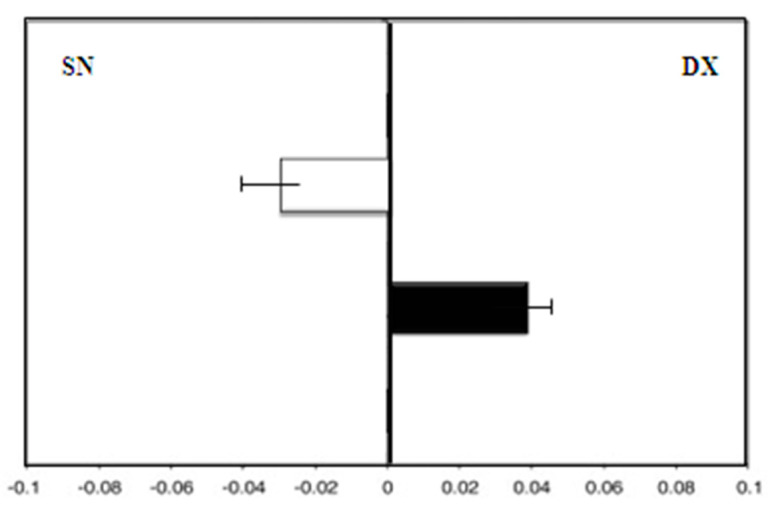
Group A (control group): mean deviations (± SD) from the true center (mm) in visual line bisection at zero time (in white) and after 36 months (in black) with the dominant right hand. Negative values reflect a deviation to the left of the objective middle, and positive values reflect a deviation to the right of the objective middle.

All patients completed the study protocol, and no side effects were reported. There were also no consistent effects related to MHT or soy isoflavones or in the control group after 18 months. Group A, the control, showed a leftward deviation (F = −3.0) at zero time. On the contrary, after 36 months, the test results showed a rightward deviation (F = 4.5; *p* < 0.01) (Figure 2).

**Figure 3:**
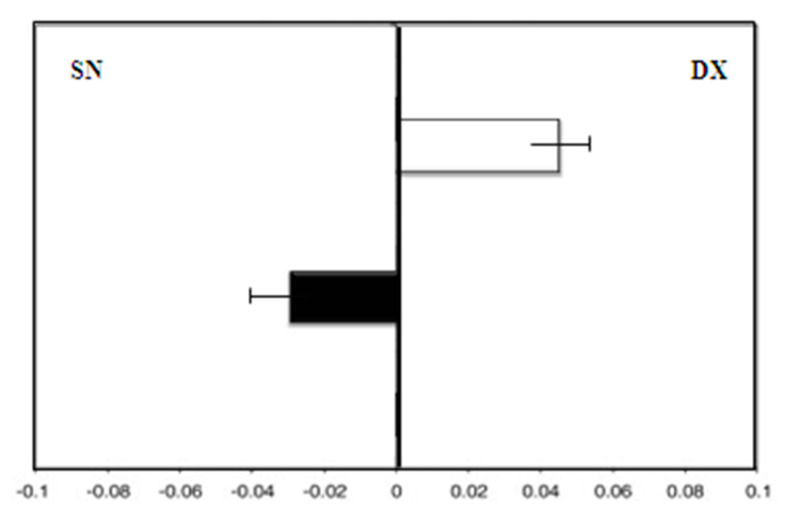
Hand-use effect in group A. Mean deviation in millimeters from the center in the line bisection test with the dominant right hand (white) and the left hand (black) at zero time.

There was greater deviation to the right with the dominant right hand (F = 4.5) than with the left hand (F = −4.0) (Figure 3).

**Figure 4:**
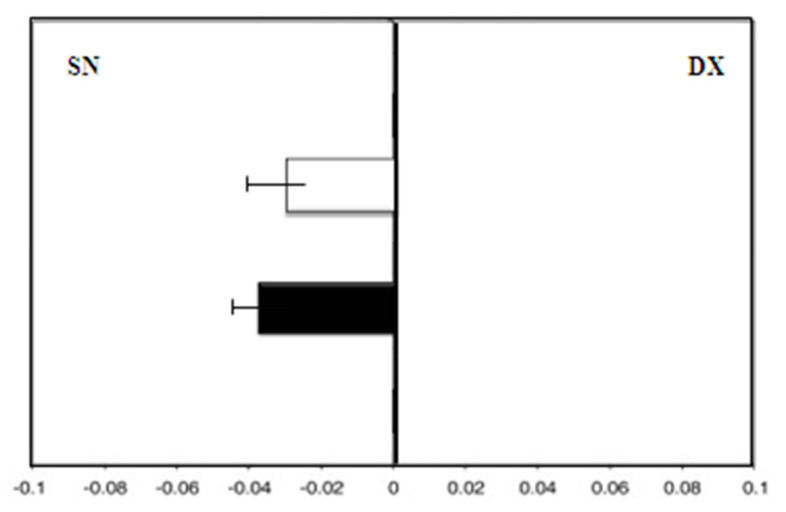
Group B: Mean deviations (± SD) from the true center (mm) in visual line bisection at zero time (in white) and after 36 months (in black) using the dominant right hand. Negative values reflect a deviation to the left of the objective middle, and positive values reflect a deviation to the right of the objective middle.

Group B (patients taking MHT), showed, at enrollment, a leftward deviation (F = −3.0). After 36 months of therapy, there was still a leftward deviation (F = −4.0; *p* = 0.08) (Figure 4).

**Figure 5:**
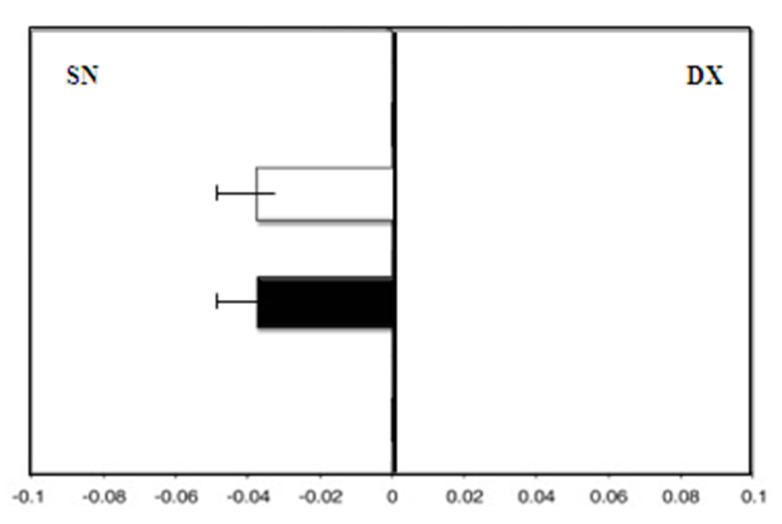
Hand-use effect in group B. Mean deviation in millimeters from the center in the line bisection test with the dominant right hand (white) and the left hand (black) at zero time.

Therefore, the hand-use effect was reduced: We observed the same deviation to the left with the dominant right hand and with the left hand (F right hand = −4.0) (Figure 5).

**Figure 6:**
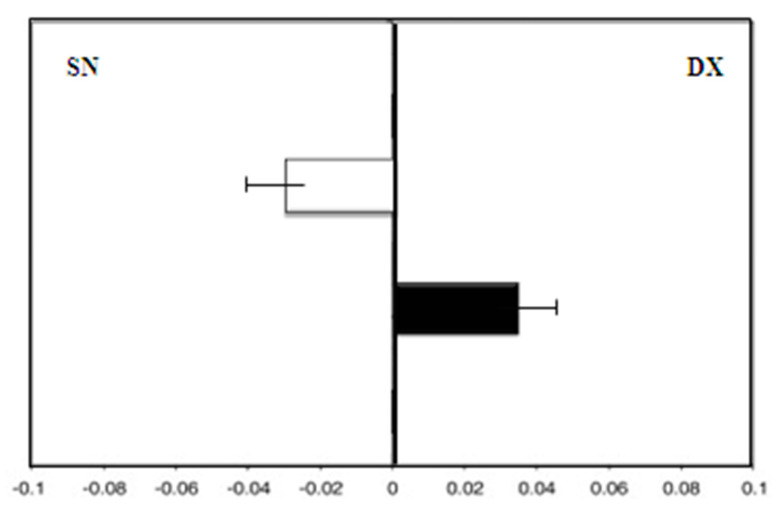
Group C (soy isoflavones group): Mean deviations (± SD) from the true center (mm) in visual line bisection at zero time (in white) and after 36 months (in black) using the dominant right hand. Negative values reflect a deviation to the left of the objective middle, and positive values reflect a deviation to the right of the objective middle.

The women taking soy isoflavones, group C, showed a leftward deviation at enrollment (F = −3.0). There was a rightward deviation (F = 3.0), with a significant difference found after 36 months (*p* < 0.01) (Figure 6).

**Figure 7:**
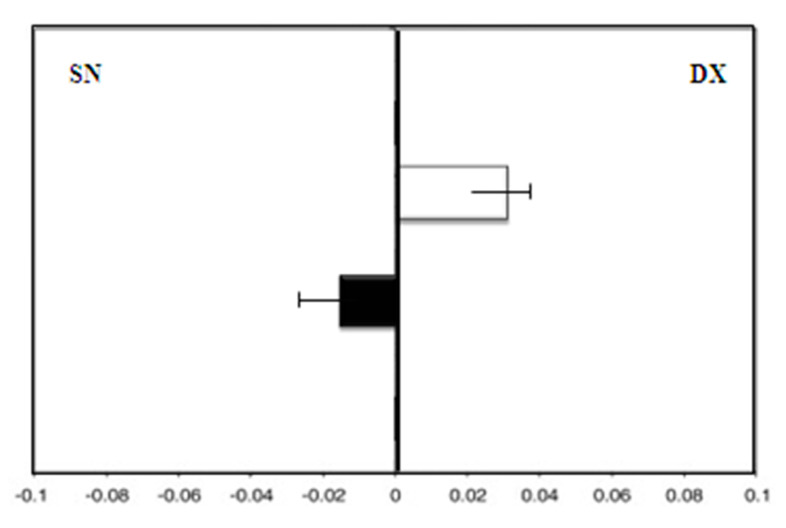
Hand-use effect in group C. Mean deviation in millimeters from the center on the line bisection test with the dominant right hand (white) and the left hand (black) at zero time.

The hand-use effect presented a rightward deviation with the dominant right hand (F = 3.0) and a leftward deviation with the left hand (F = −1.8; *p* = 0.01) (Figure 7).

Multivariate analysis was performed to investigate the role of major covariates in changing the results of the Bisiach test after 36 months. No significant influences were reported (Table 2).

## 4. Discussion

This prospective study found that postmenopausal women without MHT show a rightward deviation on the Bisiach line bisection test, reflecting typical cerebral lateralization. These results are in line with data from other authors who assessed significant asymmetries in postmenopausal women without MHT [1,6] and suggest that low levels of sexual hormones during the postmenopausal period might be associated with a “male-like” model: it features a predominance of left hemisphere activity and a reduction in the performance of the right hemisphere. In fact, since the interruptions of the left brain are not related to symptoms of negligence, several studies have hypothesized that the right hemisphere spatially represents both the contralateral and ipsilateral visual scene [23,24,25,26,27,28]. In contrast, the left hemisphere represents only the right, contralateral, side, which should therefore confirm a right-brain dominance in visuospatial processing [4,7,25]. This explains how dysfunctions of the right hemisphere lead to less efficient spatial processing, especially of the left hemifield, and a loss of inhibition in the left hemisphere, causing a better or compensated visualization of the right hemifield and, consequentially, a deviation to the right of the bisection line, as happens in patients with right brain damage.

These data highlight a functional inefficiency in the right hemisphere with the advancing of age and support the theory that age-related cognitive decline influences the functions attributed to the right hemisphere to a greater extent than those associated with the left hemisphere [7,26,27]. The validity of this hypothesis has also been confirmed in previous studies on other lateral functional domains, such as the affective domain and sensorimotor and verbal–spatial processing [23,24,25].

The data from our study also suggest an influence of the corpus callosum. Right-handed women in menopause without MHT deviate less to the right with their left hands than with their right hands (F right hand = 4.5; F left hand = −4.0), and this decrease in age-related lateralization could reflect a reduction in age-related interhemispheric inhibition due to the deterioration of the corpus callosum. It has been proven that healthy young women who have anatomical and functional integrity in the corpus callosum show a relevant leftward deviation using the dominant right hand to bisect the line because of communication between the right hemisphere, which dominates visuospatial attention, and the left hemisphere, which mainly controls the response of the right hand [19]. This deviation to the left in right-handed women is therefore interpreted as a sign of efficient interhemispheric communication and avoiding the hand-use effect. 

Permanent and stable reduction in estrogen and progesterone levels in postmenopausal women could stabilize FCA. Data regarding the mechanism of action of estrogens are still conflicting: some authors have hypothesized an effect based on a hormonal modulation of global basal brain activity by lowering the threshold for phasic activation induced by a given task [29]. However, this has a general effect on both hemispheres. Instead, the improvement in the performance of the right hemisphere is thought to reflect a specific modulation of neuronal circuits in the right hemisphere. MHT may induce neuromorphological changes in specific brain structures and, therefore, in related functions [6]. However, we speculate that MHT can regulate other functions dominated by the right hemisphere.

Previous studies investigated the variations in FCA in young women in different phases of their hormonal cycle and showed an attenuation of the related progesterone asymmetries, generally during the luteal phase [1,6]. It could be interesting to assess the impacts on FCA of both different progestins in MHT and sequential MHT regimens vs. combined ones. 

In women treated with phytoestrogens, the rightward deviation persists, although to a lesser degree. Although no significant data demonstrate a relationship between phytoestrogens and brain functions, we propose that soy isoflavones, due to their estrogen-like structure, interact with the estrogen receptors available in the brain to trigger a weak agonist effect with subsequent weak estrogenic action. However, their effect on improving FCA in spatial attention seems not to be relevant.

Despite the above, however, our study presents several limitations, such as the small sample size and the observational design. Further studies, especially randomized controlled trials, are needed to investigate the role of MHT in FCA in spatial attention better.

## 5. Conclusions

Our results suggest that the functions of the right hemisphere are mainly affected by aging, and this is perhaps one of the reasons why the right hemisphere is more susceptible to the effects of MHT. With the current indications that suggest a maximum recommended duration of MHT of five years due to the specific cardiovascular and breast risks associated with its use, the link between the duration of exposure to hormonal therapy and the duration of brain performance remains to be clarified, as does the question of whether the beneficial effects on lateralized cognitive functions persist even after suspension.

## Figures and Tables

**Table 1:** Main characteristics of the patients enrolled in the study.

Variables		Group A (*n* = 30)	Group B (*n* = 30)	Group C (*n* = 30)	*p*-Value
**Age (year)**	Mean (SD)	53.5 (1.2)	52.9 (1.5)	53.0 (1.4)	0.20
**BMI (kg/m^2^)**	Mean (SD)	23.5 (0.9)	23.4 (1.3)	23.6 (1.2)	0.79
**Time after menopause (year)**	Mean (SD)	2.3 (1.2)	2.0 (1.5)	3.5 (1.4)	0.01
**Duration of treatment (months)**	Mean (SD)	15.5 (6.2)	14.7 (5.6)	15.1 (4.4)	0.85
**Education**	Primary school	4	6	6	0.99
	Middle school	9	8	8	
	High school	11	10	10	
	University	6	6	6	

*Note*. A *p*-value < 0.05 was considered statistically significant.

**Table 2:** Multivariate analysis of factors related to influence on Bisiach’s test. All three groups were compared in the same multiple linear regression, with group A (control group) as the reference group.

Variables		Group A (*n* = 30)	*p*-Value	Group B (*n* = 30)	*p*-Value	Group C (*n* = 30)	*p*-Value
**Age (year)** >55 years	HR (95% CI)	1.44 (0.85–1.86)	0.41	1.34 (0.89–1.48)	0.56	1.14 (0.75–1.26)	0.36
**BMI (kg/m^2^)** >24 kg/m^2^	HR (95% CI)	1.09 (0.67–1.16)	0.17	1.23 (0.77–1.46)	0.24	1.17 (0.60–1.44)	0.41
**Time after menopause (years)** >2 years	HR (95% CI)	1.12 (0.59–1.24)	0.46	1.19 (0.79–1.35)	0.19	1.22 (0.88–1.44)	0.34
**Duration of treatment (months)** >15 months	HR (95% CI)	1.03 (0.87–1.11)	0.20	1.30 (0.81–1.44)	0.23	1.16 (0.97–1.31)	0.11

HR: hazard ratio; CI: confidence interval.

## Data Availability

The data presented in this study are available on request from the corresponding author.

## References

[1] Hausmann M. (2005). Hemispheric asymmetry in spatial attention across the menstrual cycle. Neuropsychologia.

[2] Duboc V., Dufourcq P., Blader P., Roussigné M. (2015). Asymmetry of the Brain: Development and Implications. Annu. Rev. Genet..

[3] Ocklenburg S., Güntürkün O. (2018). Brain Asymmetries—Two Millennia of Speculation, Research and Discoveries. The Lateralized Brain.

[4] Nadeau S.E. (2010). Hemispheric asymmetry: What, why, and at what cost?. J. Int. Neuropsychol. Soc..

[5] Brown H.D., Kosslyn S.M. (1993). Cerebral lateralization. Curr. Opin. Neurobiol..

[6] Hausmann M., Güntürkün O. (2000). Steroid fluctuations modify functional cerebral asymmetries: The hypothesis of progesterone-mediated interhemispheric decoupling. Neuropsychologia.

[7] Wisniewski A. (1998). Sexually-dimorphic patterns of cortical asymmetry, and the role for sex steroid hormones in determining cortical patterns of lateralization. Psychoneuroendocrinology.

[8] Hampson E. (1990). Variations in sex-related cognitive abilities across the menstrual cycle. Brain Cogn..

[9] McEwen B.S., Milner T.A. (2017). Understanding the broad influence of sex hormones and sex differences in the brain. J. Neurosci. Res..

[10] Cicinelli E., De Tommaso M., Cianci A., Colacurci N., Rella L., Loiudice L., Cicinelli M.V., Livrea P. (2011). Oral contraceptive therapy modulates hemispheric asymmetry in spatial attention. Contraception.

[11] Maki P., Hogervorst E. (2003). HRT and cognitive decline. Best Pract. Res. Clin. Endocrinol. Metab..

[12] De Franciscis P., Colacurci N., Riemma G., Conte A., Pittana E., Guida M., Schiattarella A. (2019). A Nutraceutical Approach to Menopausal Complaints. Medicina.

[13] De Franciscis P., Grauso F., Luisi A., Schettino M.T., Torella M., Colacurci N. (2017). Adding Agnus Castus and Magnolia to Soy Isoflavones Relieves Sleep Disturbances Besides Postmenopausal Vasomotor Symptoms-Long Term Safety and Effectiveness. Nutrients.

[14] De Franciscis P., Guida M., Schiattarella A., Riemma G., Colacurci N. (2022). Safety of non-hormonal medications for managing hot flashes. Expert Opin. Drug Saf..

[15] De Franciscis P., Conte A., Schiattarella A., Riemma G., Cobellis L., Colacurci N. (2020). Non-hormonal Treatments For Menopausal Symptoms and Sleep Disturbances: A Comparison Between Purified Pollen Extracts and Soy Isoflavones. Curr. Pharm. Des..

[16] Riemma G., Schiattarella A., La Verde M., Zarobbi G., Garzon S., Cucinella G., Calagna G., Labriola D., De Franciscis P. (2019). Efficacy of Low-Dose Paroxetine for the Treatment of Hot Flushes in Surgical and Physiological Postmenopausal Women: Systematic Review and Meta-Analysis of Randomized Trials. Medicina.

[17] Wang T., Liu Y., Zhuang X., Luan F., Zhao C. (2020). The Interaction of Isoflavone Phytoestrogens with ERα and ERβ by Molecular Docking and Molecular Dynamics Simulations. Curr. Comput.-Aided Drug Des..

[18] Bisiach E., Capitani E., Colombo A., Spinnler H. (1976). Halving a horizontal segment: A study on hemisphere-damaged patients with cerebral focal lesions. Schweiz. Arch. Fur Neurol. Neurochir. Und Psychiatr. = Arch. Suisses Neurol. Neurochir. Psychiatr..

[19] Hausmann M., Waldie K.E., Corballis M.C. (2003). Developmental changes in line bisection: A result of callosal maturation?. Neuropsychology.

[20] Kurth F., Spencer D., Hines M., Luders E. (2018). Sex differences in associations between spatial ability and corpus callosum morphology. J. Neurosci. Res..

[21] Genc S., Malpas C.B., Ball G., Silk T.J., Seal M.L. (2018). Age, sex, and puberty related development of the corpus callosum: A multi-technique diffusion MRI study. Brain Struct. Funct..

[22] Oldfield R.C. (1971). The assessment and analysis of handedness: The Edinburgh inventory. Neuropsychologia.

[23] Ruch W., Kohler G., Van Thriel C. (1996). Assessing the “humorous temperament“: Construction of the facet and standard trait forms of the State-Trait-Cheerfulness-Inventory—STCI. Humor-Int. J. Humor Res..

[24] von Elm E., Altman D.G., Egger M., Pocock S.J., Gøtzsche P.C., Vandenbroucke J.P. (2007). The Strengthening the Reporting of Observational Studies in Epidemiology (STROBE) statement: Guidelines for reporting observational studies. Lancet.

[25] Doty R.L., Kisat M., Tourbier I. (2008). Estrogen replacement therapy induces functional asymmetry on an odor memory/discrimination test. Brain Res..

[26] Dolcos F., Rice H.J., Cabeza R. (2002). Hemispheric asymmetry and aging: Right hemisphere decline or asymmetry reduction. Neurosci. Biobehav. Rev..

[27] Cabeza R. (2002). Hemispheric asymmetry reduction in older adults: The HAROLD model. Psychol. Aging.

[28] Piefke M., Onur Ö.A., Fink G.R. (2012). Aging-related changes of neural mechanisms underlying visual-spatial working memory. Neurobiol. Aging.

[29] McCourt M. (1999). Visuospatial attention in line bisection: Stimulusmodulation of pseudoneglect. Neuropsychologia.

